# Effectiveness of Health Information Package Program on Knowledge and Compliance among Pregnant Women with Anemia: A Randomized Controlled Trial

**DOI:** 10.3390/ijerph19052724

**Published:** 2022-02-26

**Authors:** Nadia B. Elsharkawy, Enas M. Abdelaziz, Marwa M. Ouda, Fatma A. Oraby

**Affiliations:** 1 Department of Nursing, College of Applied Medical Sciences, Jouf University, Sakaka 72388, Jouf, Saudi Arabia; emabdelhamid@ju.edu.sa (E.M.A.); mmouda@ju.edu.sa (M.M.O.); 2 Department of Maternal and Newborn Health Nursing, Faculty of Nursing, Cairo University, Cairo 11562, Egypt; 3 Department of Psychiatric Mental Health Nursing, Faculty of Nursing, Cairo University, Cairo 11562, Egypt; 4 Department of Pediatric Nursing, Faculty of Nursing, Damanhour University, Damanhour 22516, Egypt; 5 Department of Obstetrics and Gynecology Nursing, College of Nursing-Misr University for Science and Technology, 6th of October City 12566, Egypt; fatma.oraby@must.edu.eg

**Keywords:** anemia, pregnant women, knowledge, compliance, education program

## Abstract

Despite the availability of iron supplements during pregnancy for all pregnant women receiving antenatal care in Saudi Arabia, anemia remains to be a global public health concern leading to adverse maternal, fetal, and neonatal effects. We aimed to evaluate the effectiveness of the Health Information Package Program on the knowledge anemic pregnant women had about anemia, their compliance with iron and folic acid supplementation, and their hemoglobin levels. A single-blind randomized controlled trial was carried out in accordance with the Consolidated Standards of Reporting Trials (CONSORT) guidelines between January and May 2021. Pregnant women (n = 196) aged 18–45 years old and diagnosed with anemia during the first trimester of their pregnancy were randomly assigned into two groups: the intervention group (Health Information Package Program plus routine care, n = 98) and the control group (routine care only, n = 98). Knowledge, the ability to select appropriate food, and hemoglobin levels were assessed at baseline and after three months, while compliance with iron and folic acid supplementation was also measured at the end of three months. This study indicated that the post-education mean knowledge score, food selection ability score, compliance rate, and hemoglobin level were significantly higher for the intervention group than for the control group. The Health Information Package Program with regular follow-ups using the WhatsApp platform was an effective educational intervention for anemic pregnant women.

## 1. Introduction

Anemia is a global health concern affecting women of reproductive age, pregnant women, adolescent girls, and young children from low- and middle-income countries [1,2,3]. It is defined as a low hemoglobin (Hb) concentration or low red blood cell count that does not meet the physiological needs of the individual [1,2,4]. Iron deficiency anemia (IDA) is the most common type of anemia and has the highest prevalence. It occurs when nutritional iron intake is insufficient for Hb synthesis [5]. Worldwide, it is estimated that 40% of all pregnant women are anemic, with a prevalence rate of 22.6% to 63% in the Eastern Mediterranean Region (EMR) and 45.5% in Saudi Arabia [2,6,7]. According to [8], the rate of anemia among pregnant women in Saudi Arabia was 53% in 2019, highlighting a severe national health concern. 

Anemia during pregnancy can increase the risk of serious health consequences for both the mother and the neonate, including abortion, stillbirth, preterm labor, intrauterine growth restriction, low birth weight, postpartum bleeding, infection, and mortality [2,9,10,11]. Risk factors associated with anemia among pregnant women include short interpregnancy intervals, teenage pregnancy, low educational level, multiparity, low meat consumption, heavy menstrual bleeding (with or without blood clots), personal or family history of anemia, and the use of non-steroidal anti-inflammatory drugs (NSD) and anti-acids [12,13].

To tackle this problem, it has been recommended that a daily oral iron supplementation of 30–60 mg of elemental iron and 400 µg of folic acid (IFA) is provided from the second trimester as a routine part of antenatal care [1]. This aims to improve the Hb levels and iron status of pregnant women and to lower the risk of anemia, puerperal sepsis, low birth weight, preterm birth, and neonatal neural tube defects [6,14]. Systematic reviews have demonstrated that consuming at least 90 iron-containing supplements during pregnancy can reduce anemia during pregnancy by up to 70% [15,16].

Although iron supplements are provided free of charge in most countries, the major problem is poor or low compliance [15,16]. This could be due to personal behaviors, such as forgetfulness, the unpleasant taste, cultural issues, environmental factors, a lack of awareness, socio-demographic status, the fear of side effects, the inadequate use of prenatal healthcare services, and poor counseling from healthcare providers regarding the proper intake of IFA tablets [17,18,19,20]. A study conducted in Saudi Arabia showed a suboptimal iron intake among 77% of pregnant women and a suboptimal folic acid intake among 64% of pregnant women [21]. Non-compliance with iron supplements could have negative consequences on pregnant women and their fetal health, and increased compliance to iron supplementation has been linked with a lower risk of anemia during pregnancy and hemorrhagic disease in the newborn [20].

The Health Information Package Program (HIPP) is a structured, planned health education program that was developed by [22], which was then was adopted and translated into simple, understandable Arabic language that is appropriate for Arab culture by [23]. The program has proved its efficacy among pregnant women by increasing their awareness of anemia, enhancing their compliance with iron supplementation, raising their Hb levels, and increasing their ability to choose iron-, vitamin C-, and protein-rich food [22,23].

There is a need for immediate and appropriate intervention with proper education to lower the rates of anemia among pregnant women. The introduction of health education for anemic pregnant women has not been established in Saudi Arabia. Less attention is paid to the awareness of food sources that are rich in iron, vitamin C, and protein and that contribute toward iron supplementation or increasing iron absorption. From an extensive literature review in Saudi Arabia, researchers identified a gap in the research regarding the efficacy of a health education intervention for pregnant women with anemia. We aim to evaluate the effectiveness of the HIPP on anemic pregnant women’s knowledge about anemia, compliance with IFA supplementation, and Hb levels. The researchers hypothesized that the HIPP could be more effective than only using routine antenatal care in improving knowledge about anemia, compliance with iron supplementation, and the levels of Hb among pregnant women with anemia after three months of intervention. 

## 2. Materials and Methods

### 2.1. Design and Participants

A single-blind randomized controlled trial with two parallel groups was conducted at an antenatal outpatient clinic at a primary healthcare center in the Sakaka Al-Jouf region of Saudi Arabia between January and May 2021. The primary outcome measure was an improvement in the hemoglobin levels of anemic pregnant women, and the secondary outcomes included improvements in knowledge, the ability to select appropriate food, and compliance with IFA supplements. The sample size was calculated using G-Power software based on the study by [23], considering 90% power and 95% confidence interval. The total required sample was 178 anemic pregnant women; this number was increased to 196 to account for the expected attrition rate of 10%. Participants were randomly allocated to either the intervention group or the control group (n = 98 in each group) with an allocation ratio of 1:1 made by an independent biostatistician. Permutation block randomization was performed to validate the equal distribution between the groups. Randomization was ensured by placing the numbers in a sealed opaque envelope, and all participants were blind to their group allocation. The Consolidated Standards of Reporting Trials (CONSORT) was considered as the guidance for the study randomization (see Supplementary Material File S1).

The inclusion criteria were as follows: (a) pregnant women diagnosed with anemia during the first trimester of their pregnancy; (b) a Hb level of <11 gm/dL and hematocrit < 30%; (c) aged 18–45 years old; (d) 14–16 weeks of gestation; (e) single fetus; and (f) have access to a smartphone with the WhatsApp application. The participants were excluded if: (a) they could not read or write; (b) multiple feti; (c) suffering from hereditary anemia or chronic medical illnesses, such as cardiac and renal diseases, or had a history of psychiatric illness; and (d) pregnant women with a Hb level of <7 gm/dL and those already on IFA supplementation for longer than one week.

### 2.2. Instruments

A four-sectioned structured interview questionnaire was used to collect data from the study participants based on the study objectives. Permission and acceptance for using the Arabic version of the tool were obtained from [23]. The tool had an internal reliability for the Arabic version of Cronbach alpha = 0.938 and the content validity index (CVI) was 0.91, which are considered high for both tool reliability and validity. This Arabic version was tested for structure, clarity, applicability, and the time needed to complete the questionnaire in a pilot study with 10 participants in each group.

#### 2.2.1. Socio-Demographic and Health-Related Information 

The questionnaire involved questions about the participants’ age, education, occupation, family monthly income in Saudi Riyal (SAR, which equals EUR 0.23), pregnancy intervals, parity, the regularity of antenatal follow-up, and the source of the heir health information about pregnancy.

#### 2.2.2. Structured Knowledge Interview Schedule (SKIS)

Participants’ knowledge about anemia during pregnancy was assessed at baseline and after three months using 21 general knowledge questions about the concept of anemia in pregnancy, the causes, signs and symptoms of anemia, the effect of anemia during pregnancy on maternal, fetal, and neonatal health, the management of iron deficiency anemia (including iron therapy and dietary management), and the prevention of anemia during pregnancy. Each correct response received one point, while an incorrect or “I do not know” response received zero points. Multiple responses were allowed; hence, the total score for all 21 knowledge questions ranged from 0 to 71 points. Participants’ overall knowledge was categorized using modified Bloom’s cut-off point and was categorized as good when their score was between 80% and 100% (57–71 points), moderate when their score was between 50% and 79% (36–56 points), and poor when their score was less than 50% (<36 points). The reliability score was 0.823 (Noronha et al., 2013). In this study, this questionnaire had a good internal reliability (Cronbach’s α value = 0.79). 

#### 2.2.3. The Food Selection Ability Checklist

The food selection ability checklist included three checklists regarding foods that are rich and poor in iron sources (17 items), vitamin C (15 items), and protein (21 items). Out of all 53 food selection ability items, only 30 correct items were selected, including food that is rich in iron (13), vitamin C (6), and protein (11). Participants’ overall food selection ability was classified as good when their score was ≥75% (23–30 items), moderate when their score was between 50% and 75% (15–22 items), and poor when their score was ≤50%, (<15 items). This tool had a reliability score of 0.706.

#### 2.2.4. Compliance with IFA Supplementation

The questionnaire also included questions related to the extent to which the pregnant women took IFA supplements as prescribed by their healthcare provider. During the three-month study period, the maximum number of IFA tablets that could have been taken was 90, based on a daily consumption of one tablet of IFA supplementation starting from the 16th week of gestation. The pregnant women were divided into three categories based on their compliance [19,24]: high compliance was 68–90 tablets (≥75%); partial compliance was 45–67 tablets (50%–75%) tablets; and non-compliance or poor compliance was less than 45 tablets (<50%). The causes of compliance or non-compliance section of the questionnaire also included questions related to the gastrointestinal side effects that can occur when taking IFA tablets, such as vomiting, constipation, flatulence, headaches, and taste impairment, and the lack of awareness regarding the importance of IFA supplementation. The tool had a strong reliability score of 0.904.

### 2.3. Intervention

Individual education was delivered to the intervention group for 30 min using a PowerPoint presentation based on the HIPP. The presentation consisted of information about anemia during pregnancy, including: definitions, risk factors, causes of anemia, the signs and symptoms of anemia, adverse effects of anemia on mothers, fetus, and neonates, and information regarding IFA supplementation guidelines. The guidelines include information about taking iron supplements regularly, how to achieve the better absorption of iron supplements, the side effects of iron supplements, and food that is rich in iron, vitamin C, and protein and can enhance iron absorption. Educational brochures were also distributed to the participants at the end of the education session. An opportunity was provided for the participants to discuss the education content and clarify any confusion. Content validity for the education session was assured by sending its content to three experts in maternity nursing.

From the 16th week of gestation and for the three-month (90-day) study period, each participant in the intervention group received one educational health message and four reminders in Arabic every week to make sure they were taking the iron supplements. The messages were sent through the WhatsApp platform, as a technological method that is helpful for delivering interventions to improve and retain acquired knowledge, until the 28th week of gestation. To ensure that each message was delivered, participants were asked to send an empty response message after receiving each WhatsApp message. To further ensure compliance with the intervention, the participants were asked to send at least three feedback messages regarding their understanding of the information content in the educational messages and to raise any queries that they had. 

The iron supplementation was provided to all participants in the intervention and the control groups every month (30 iron capsules with 150 mg of ferrous sulfate and 0.5 mg of folic acid). They received their repeat prescription through “Wasfaty”, a service provided by the Saudi Arabian Ministry of Health that allows patients to receive medications from the nearest pharmacy registered in the private sector through an e-platform that connects primary healthcare centers. The pregnant women could also receive their prescriptions during routine antenatal visits. All participants received information regarding the proper usage of iron therapy and were asked to start iron supplementation by 16 weeks of gestation to assess their compliance. 

The participants in the control group received routine antenatal care, including: vital sign checks; weight, blood, and urine tests; and physical examinations. For ethical consideration, the participants in the control group were able to receive the HIPP education after finishing the study.

### 2.4. Data Collection

After obtaining official approval to conduct the study and written informed consent from the participants, and before starting the intervention, the researchers interviewed all eligible participants using face-to-face structured interviews at the previously mentioned clinical setting to complete the study questionnaires. Only the compliance questions could not be determined in the pretest as they were based on the total number of iron tablets consumed over the three months. All participants were asked to answer all questions (baseline assessment). The interviews took around 20 to 30 min each, fixed time, in a specified room in the antenatal clinic used for the program. All participants received the study questionnaires in the same clinical settings three months later (at 28 weeks of gestation). As a part of standard antenatal care, all pregnant women had a complete blood count (CBC) assessment that was repeated every three months and at the time of delivery. Hb level was collected from each participant’s antenatal care record at baseline and three months after recruitment. Data were collected through the period of January to May 2021.

### 2.5. Statistical Analysis

Data were fed into the computer and analyzed using the IBM SPSS software package, version 20.0 (IBM Corp., Armonk, NY, USA). Data were presented in the form of descriptive statistics, such as frequency, percentages, mean, and standard deviation. The Kolmogorov–Smirnov test was used to verify the normality of distribution. The categorical variables were compared to the Chi-squared test, while for the independent samplea t-test was used to compare the two independent groups with normal distributions. The Mann–Whitney U test was performed to compare data with non-normal distributions. The Wilcoxon signed-rank test was used for abnormally distributed quantitative variables to compare the measurements taken at the baseline and after three months. the effect size (Cohen’s D) was used to measure the significance of the intervention effect. It was categorized as follows: <0.2 was no effect, 0.2–<0.5 was a small effect, 0.5–<0.8 was a medium effect, and >0.8 was a significant effect that indicated a strong relationship between the two variables [25]. Regression was performed to detect the most independent variables affecting compliance. The results were considered significant at *p* < 0.05.

### 2.6. Ethical Consideration

The local bioethics committee approved the study protocol (IRB number 15–04–42) and the trial was registered on the ClinicalTrials.gov (accessed on 17 June 2021) registry website with clinical trial identifier NCT04661865. The study objectives were explained to all participants and written informed consent was obtained. Participants were also informed that they had the right to withdraw from the study at any time with no adverse consequences. Code numbers were created and the confidentiality of data was maintained.

## 3. Results

**Figure 1:**
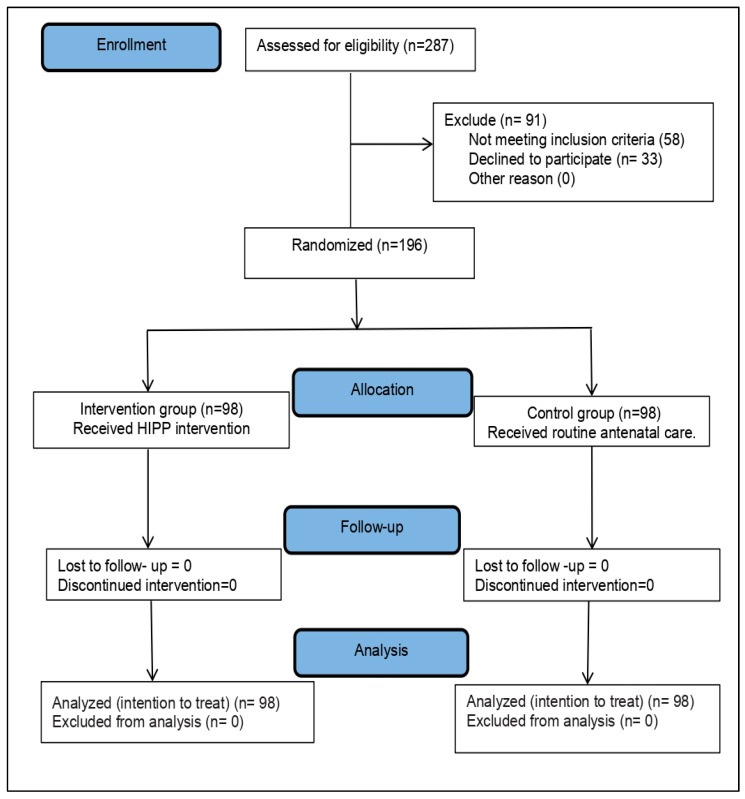
A consort flow diagram of the study.

Initially, 287 pregnant women with anemia were screened for eligibility during data collection. In total, 58 women were excluded from the study because they did not meet the inclusion criteria and 33 women refused to participate. Finally, 196 women were randomly assigned into the intervention group (98) and the control group (98). Figure 1 summarizes the study flow diagram.

There were no statistically significant differences between the socio-demographic characteristics of participants in the control group and those in the intervention group in terms of age, educational level, occupation, family monthly income, parity, pregnancy intervals, and antenatal follow-ups (*p* > 0.05; Table 1). 

Before the intervention, there were no significant differences between the two groups in the mean knowledge score (*p* < 0.070), food selection ability score (*p* = 0.410), or Hb levels (*p* = 0.584). After the three-month intervention, there was a significant difference between the intervention and control groups in terms of the mean score and effect size of knowledge, food selection ability, and Hb levels (*p* < 0.001), indicating a large effect size for Hb level (d = 0.850) and a medium effect size for knowledge and food selection ability (d = 0.773 and 0.617, respectively) (Table 2).

Table 3 shows the mean scores for taking iron tablets for the intervention group (82.31 ± 8.71) and the control group (66.78 ± 13.82). The majority of the participants in the intervention group (90.8%) had a high compliance with taking the IFA supplements compared to the control group (66.4%), with a statistically significant difference between the two groups (*p* < 0.001).

Univariate and multivariate linear regression analyses were conducted to identify the variables that could independently predict higher compliance rates among the participants of the intervention group following the program. Younger age, higher educational level (university and postgraduates), primiparity, regular antenatal follow-up visits, high knowledge score, and good food selection ability were independently associated with high compliance. The most identified factors that affected compliance were higher educational levels (*p* = 0.001; Table 4).

## 4. Discussion

This study evaluated the effectiveness of the HIPP on improving anemic pregnant women’s knowledge about anemia, compliance with IFA supplementation, and Hb levels. It was observed that the anemic pregnant women who received HIPP in the form of individual educational intervention using a PowerPoint presentation, brochures, and follow-up educational health messages and reminders using WhatsApp had a significant increase in the mean knowledge score, food selection ability score, Hb level, and compliance with IFA supplements compared to those in the control group. Similarly, studies from Jordan, India, and Indonesia also found an improvement in the mean knowledge score, food selection ability score, Hb level, and compliance rate following the education program [5,22,23].

At the end of the intervention, there was a significant difference in Hb levels between the intervention and control groups (*p* < 0.001), emphasizing the effect of education intervention in improving Hb levels. This result was consistent with that of the study by [26], who stated that health education improves the Hb status among pregnant Malaysian women. Similarly, a quasi-experimental study conducted in Nepal found that nutritional education emphasizing iron-rich food consumption was positively associated with higher Hb levels [27]. Ref. [19] reported improved Hb levels and higher compliance with iron supplements among pregnant Iranian women after 12 weeks of education using SMS messaging.

On the other hand, [28] observed higher Hb levels in the intervention group than the control group; however, the t-test showed no statistically significant difference (t = −0.68, *p* = 0.620) 12 weeks after the administration of a culturally tailored nutritional education intervention to pregnant Omani women. The mean difference in Hb levels between the intervention and control groups was more than 1.0 g/dL. This difference was mainly based on the higher IFA supplementation uptake, reflecting the increase in the compliance rate among women in the intervention group compared to the control group that was also found in other studies [19,26,28,29].

We found that the mean knowledge score for the intervention group was higher than that for the control group (*p* < 0.001) with a mean difference of 29.18, reflecting a gain in knowledge with a medium effect from the educational intervention. Similar results were reported from studies conducted in Jordon, India, Indonesia, Nepal, Iran, and Malaysia, which reported that health educational interventions were able to improve knowledge levels during pregnancy after three months [5,19,22,23,26,27].

Our study also focused on dietary modifications and how education intervention could help anemic pregnant women to improve their ability to select appropriate food, take IFA supplements properly, and change unhealthy habits. Many pregnant women take IFA supplements after tea, coffee or milk, which prevents its absorption. The awareness among pregnant women of the proper use of different dietary supplements before, after, and during pregnancy needs to improve. Education is recommended for pregnant women through media and healthcare professionals. Progress is needed toward implementing national recommendations for dietary supplements during pregnancy. As is well known, the traditional Saudi diet has been replaced by an energy-dense Western diet and combined with a sedentary lifestyle, thereby increasing the prevalence of non-communicable diseases, such as obesity, type 2 diabetes mellitus, and hypertension [30].

The Ministry of Health (MOH) in Saudi Arabia has made great efforts to provide all pregnant women with everything they need for safe pregnancies and childbirth. IFA supplements are provided free of charge throughout the pregnancy, and accessible antenatal care through the “Mowid” service is a Saudi Arabian Ministry of Health application that allows patients and beneficiaries to book, cancel or reschedule appointments at primary healthcare centers, as well as manage their referrals. The MOH portal has an awareness platform that further supports pregnant women with information about pregnancy including nutrition, IFA supplementation guidelines, healthy pregnancy, healthy weight gain, etc. [31]. Posters and brochures are available in all healthcare centers and public hospitals in Saudi Arabia.

A cross-sectional study in Muntinlupa, Philippines, observed that the majority (85.6%) of pregnant women who took the IFA supplements were knowledgeable about the different aspects of IFA supplementation [32]. The RCT in Iran showed that the mean number of IFA tablets taken was 80.5 in the intervention group and 67.2 in the control group [19]. The findings of these two studies were consistent with our results, as the mean number of iron tablets taken was 82.31 ± 8.71 in the intervention group and 66.70 ± 13.82 in the control group. 

In contrast, a cross-sectional study conducted in Saudi Arabia found that the compliance rate for iron supplementation was 49.7%; however, this was seen among those with a lower educational level, age > 35 years, and having had more than six previous pregnancies [24]. A systematic review and meta-analysis in Ethiopia observed a 43.63% compliance rate with IFA supplementation [33]. A study from Aykel town, Northwest Ethiopia, revealed that 45.1% of pregnant women were knowledgeable about anemia and 47.6% adhered to IFA supplementation [34]. The results of these studies were lower than the World Health Organization’s recommendation for iron tablet intake (at least 90 tablets) and aligned with our results in that the compliance with iron supplementation was low among the women in the control group who did not receive any intervention. This study had some limitations. We did not see the pregnant women taking the IFA supplements to determine compliance but rather depended solely on self-reporting, which could have led to a response bias. It was impossible to have an actual control group for ethical reasons, which meant that IFA supplements could not be replaced with a placebo because this would have affected the pregnancy status. The effectiveness of the HIPP was mainly implemented among anemic pregnant women in Saudi Arabia and thus, the results of the studied group cannot be generalized due to the lack of representativeness. Our study’s strength was the use of a single-blinded randomized controlled trial research design with allocation concealment and the fact that the intervention could be tested in other RCTs. It also provided a novel approach in using the WhatsApp platform to improve compliance with IFA supplementation during pregnancy, potentially saving resources for the healthcare system.

## 5. Conclusions

The Health Information Package Program (HIPP) with follow-up educational health messages and reminders using the WhatsApp platform was an effective intervention for anemic pregnant women in Saudi Arabia. It helped to improve the women’s knowledge regarding anemia during pregnancy, increase the women’s awareness of better food selection, enhance their compliance with iron supplementation, and increase their Hb levels. The Health Information Package Program should be endorsed by policymakers and used as a comprehensive national strategy to prevent anemia during pregnancy. Nurses, midwives, and obstetricians could use the Health Information Package Program as an effective educational intervention among pregnant women, which is evidenced by the improvement in hemoglobin levels. Further and more detailed surveys on anemia during pregnancy and its associated factors should be conducted to ensure the representativeness of the results to the population.

## Figures and Tables

**Table 1:** The socio-demographic characteristics and health-related information of the study participants (n = 196).

Socio-Demographics	Total (n = 196)		Intervention (n = 98)		Control (n = 98)		Test of Sig.	*p*
	No.	%	No.	%	No.	%		
Age (Years)								
<25	98	50.0	45	45.9	53	54.1	χ^2^ = 1.777	0.411
25–34	75	38.3	42	42.9	33	33.7		
35–45	23	11.7	11	11.2	12	12.2		
Mean ± SD	25.93 ± 5.30		25.76 ± 5.42		26.10 ± 5.19			
Educational Level								
Primary	40	20.4	21	21.4	19	19.4	χ^2^ = 1.081	0.582
Intermediate and Secondary	47	24.0	26	26.5	21	21.4		
University and Postgraduates	109	55.6	51	52.0	58	59.2		
Occupation								
Housewife	99	50.5	54	55.1	45	45.9	χ^2^ = 1.670	0.434
Working	47	24.0	21	21.4	26	26.5		
Students	50	25.5	23	23.5	27	27.6		
Family Monthly Income								
Poor: <SAR 5000 (<EUR 1170)	10	5.1	7	7.1	3	3.1	χ^2^ = 4.583	0.101
Average: SAR 5000 < SAR 1000 (EUR 1170 < EUR 2340)	128	65.8	68	69.4	60	61.2		
Good: ≥SAR 1000 (≥EUR 2340)	58	29.6	23	23.5	35	35.7		
Parity							χ^2^ = 1.074	0.783
Nullipara	74	37.8	40	40.8	34	34.7		
Primipara	74	37.8	36	36.7	38	38.8		
Multipara	30	15.3	13	13.3	17	17.3		
Grand Multipara	18	9.2	9	9.2	9	9.2		
Pregnancy Intervals							χ^2^ = 0.330	
Short: <2 years	108	55.1	56	57.1	52	53.1		0.566
Adequate: ≥2 years	88	44.9	42	42.9	46	46.9		
Antenatal Follow-up	146	74.5	76	77.6	70	71.4	χ^2^ = 0.967	0.326

χ^2^, Chi-squared test; *p*, *p* value for comparing between the studied groups.

**Table 2:** A comparison between participants’ mean scores for knowledge, food selection ability, and Hb levels.

Variable	Outcome	Pretest	Posttest	Mean Difference	Z	*p*	Effect Size Eta	Level
		Mean (SD.)	Mean (SD.)					
Knowledge (0–71)	Intervention	25.14 ± 10.13	54.33 ± 10.92	29.18	8.201 *	<0.001*	0.773	Intermediate
	Control	23.55 ± 8.15	24.79 ± 10.19	1.23	0.527	0.598	0.012	No effect
	U (p0)	4217.0 (0.070)	441.5 * (<0.001 *)					
Food Selection Ability (0–53)	Intervention	13.41 ± 3.99	21.80 ± 5.81	8.39	7.874 *	<0.001 *	0.617	Intermediate
	Control	12.67 ± 2.35	13.02 ± 2.90	0.35	1.298	0.194	0.019	No effect
	U (p0)	4626.500 (0.410)	677.0 * (<0.001 *)					
Hb Level	Intervention	9.96 ± 0.30	11.16 ± 0.50	1.20	8.620 *	<0.001 *	0.850	Large
	Control	9.99 ± 0.32	10.01 ± 0.37	0.02	1.841	0.066	0.037	No effect
	U (p0)	4593.500 ≥ (0.584)	245.50 * (<0.001 *)					

U, Mann–Whitney U test; Z, Wilcoxon signed-rank test; *p*, *p* value for comparing between pre- and posttest results; p0, *p* value for comparing between studied groups. * Statistically significant at *p* < 0.05.

**Table 3:** The compliance of the study participants (n = 196) with the iron and folic acid (IFA) supplementation.

Compliance	Intervention (n = 98)		Control (n = 98)		Test of Sig.	*p*
	No.	%	No.	%		
Poor < 45 (<50%)	2	2.1	17	17.3	χ^2^ = 19.104	<0.001 *
Partial 45–67 (50–74)	7	7.1	16	16.3		
High 68–90 (≥75–100%)	89	90.8	65	66.4		
Number of IFA Tablets Taken, Mean ± SD.	82.31 ± 8.71		66.78 ± 13.82		U = 1366.0 *	<0.001 *

χ^2^, Chi-squared test; *p*, *p* value for comparing between the studied groups. * Statistically significant at *p* < 0.05.

**Table 4:** Univariate and multivariate linear regression analyses for the factors affecting compliance.

Parameter	Univariate		# Multivariate	
	*p*	B (95% C.I)	*p*	B (95% C.I)
Age (Years)	0.048 *	−0.371 (−0.739–−0.003)	0.880	−0.026 (−0.365–0.313)
Educational Level	<0.001 *	6.148 (3.844–8.451)	0.001 *	4.631 (2.018–7.244)
Family Monthly Income	0.349	1.743 (−1.915–5.401)		
Parity	0.003 *	−2.991 (−4.963–−1.019)	0.893	0.317 (−4.325–4.959)
Pregnancy Interval	0.494	1.370 (−2.575–5.314)		
Antenatal Follow-up	0.009 *	−5.935 (−10.361–−1.508)	0.906	0.293 (−4.577–5.164)
Knowledge	<0.001 *	0.319 (0.257–0.381)	0.069	0.106 (−0.008–0.220)
Food Selection Ability	<0.001 *	0.639 (0.503–0.776)	0.594	0.059 (−0.277–0.159)

R^2^ = 0.486; F = 19.541; B, unstandardized coefficients; C.I, confidence interval. # All variables with *p* < 0.05 were included in the multivariate analysis. * Statistically significant at *p* < 0.05.

## Data Availability

Data are available from the corresponding author (Nadia B. Elsharkawy) upon reasonable request.

## Supplementary Materials

The following are available online at https://www.mdpi.com/article/10.3390/ijerph19052724/s1, File S1: CONSORT 2010 checklist of information to include when reporting a randomized trial.
